# Staurosporine and Extracellular Matrix Proteins Mediate the Conversion of Small Cell Lung Carcinoma Cells into a Neuron-Like Phenotype

**DOI:** 10.1371/journal.pone.0086910

**Published:** 2014-02-28

**Authors:** Tamara Murmann, Carmen Carrillo-García, Nadine Veit, Cornelius Courts, Alexander Glassmann, Viktor Janzen, Burkhard Madea, Markus Reinartz, Anne Harzen, Michael Nowak, Sven Perner, Jochen Winter, Rainer Probstmeier

**Affiliations:** 1 Neuro- and Tumor Cell Biology Group, Department of Nuclear Medicine, University Hospital of Bonn, Bonn, Germany; 2 Department of Hematology and Oncology, University Hospital of Bonn, Bonn, Germany; 3 Institute of Legal Medicine, University of Bonn, Bonn, Germany; 4 Institute of Anatomy and Cell Biology, University of Bonn, Bonn, Germany; 5 Oral Cell Biology Group, Department of Periodontology, Operative and Preventive Dentistry, Bonn, Germany; 6 Proteomics Group, Max-Planck-Institute for Plant Breeding Research, Cologne, Germany; 7 Department of Prostate Cancer Research, Institute of Pathology, University Hospital of Bonn, Bonn, Germany; Cincinnati Children's Hospital Medical Center, United States of America

## Abstract

Small cell lung carcinomas (SCLCs) represent highly aggressive tumors with an overall five-year survival rate in the range of 5 to 10%. Here, we show that four out of five SCLC cell lines reversibly develop a neuron-like phenotype on extracellular matrix constituents such as fibronectin, laminin or thrombospondin upon staurosporine treatment in an RGD/integrin-mediated manner. Neurite-like processes extend rapidly with an average speed of 10 µm per hour. Depending on the cell line, staurosporine treatment affects either cell cycle arrest in G2/M phase or induction of polyploidy. Neuron-like conversion, although not accompanied by alterations in the expression pattern of a panel of neuroendocrine genes, leads to changes in protein expression as determined by two-dimensional gel electrophoresis. It is likely that SCLC cells already harbour the complete molecular repertoire to convert into a neuron-like phenotype. More extensive studies are needed to evaluate whether the conversion potential of SCLC cells is suitable for therapeutic interventions.

## Introduction

SCLC is a highly aggressive neuroendocrine tumor [1] with an incidence rate of about 10 to 15% of all lung cancers [2]. The majority of SCLCs arises from neuroendocrine cells, although alveolar type 2 cells may also contribute [3], [4]. The expression of neuroendocrine/neural marker molecules, such as achaete-scute homologue-1 (hASH-1) NCAM180, neurofilaments, neuron-specific enolase or neurotrophin receptors is a common characteristic of SCLC cells [5], [6]. Although the initial response rate to chemo- and radiotherapy is in the range of 60 to 80%, more than 95% of patients die within five years of diagnosis. These numbers have not considerably changed during the past 30 years, when cisplatin/etopoiside in combination with radiation was introduced as a primary standard for first line therapy [7], [8]. A considerable amount of data has been collected during the last years concerning the major genetic changes present in this tumor type, i.e. loss or mutation of TP53, Rb, PTEN and PI3K, as well as amplification of members of the MYC family of oncogenes [9], [10], but this knowledge could not be transferred into successful targeted therapies.

One major issue in cancer therapy is to reduce or at best stop tumor cell proliferation. Differentiation therapy is aimed to induce in cancer cells the natural pathway of terminal differentiation or even senescence. But even if differentiation of cancer cells would not reduce proliferation it could induce the expression of new genes, which may represent therapy-relevant targets. For many years, treatment of acute promyelocytic leukemia (APML) with retinoic acid and arsenic trioxide was the prime example for a successful intervention based on differentiation processes, but at present degradation of the PML-RARA oncoprotein but not cellular differentiation per se is assumed to be the major mechanism to eradicate APML [11]. For other types of cancer, promising data have so far been provided mainly in in-vitro or in animal models, such as inhibition of proliferation along with lipid accumulation in breast cancer cells upon treatment with the PPARγ agonist troglitazone [12], interleukin-15-mediated epithelial differentiation of renal tumor stem cells [13] or antiinvasive, antiangiogenic, as well as proapoptotic effects in retinoic acid-differentiated stem-like glioma cells [14].

Against this background it appeared plausible to evaluate the capacity of SCLC cells to develop a neuronal or neuron-like phenotype. To our knowledge, only limited data are available concerning this topic. Nerve growth factor reversibly reduces the proliferative capacity and tumorgenicity in some SCLC cell lines but morphological alterations have not been reported [15]. Moreover, process formation has been demonstrated for a subset of SCLC cell lines when cultivated on a laminin (LAM) substrate, whereby their proliferation capacity remained constant [16].

Here, we show that four out of five SCLC cell lines reversibly develop a neuron-like phenotype in the presence of the unspecific PKC inhibitor staurosporine (SSP) when cultivated on an ECM substratum, such as fibronectin (FIB), LAM, and thrombospondin (THR) or on RGDS-peptides. Hence SSP induces neuron-like conversion in the range of hours the necessary molecular repertoire is most likely already expressed by unstimulated SCLC cells. A possible therapeutic relevance of these data is discussed.

## Materials and Methods

### Materials

ECM proteins: Basement membrane matrix (BME; from Life technologies) and laminin (LAM; from Roche) were prepared from mouse EHS sarcoma; FIB (from Sigma) was purified from bovine plasma and THR (from Athens Research and technology) from human platelets. Antibodies: monoclonal phospho-p44/42 MAPK antibody to dually phosphorylated ERK1/2, polyclonal p44/42 MAPK antibodies recognizing both phosphorylated and unphosphorylated ERK1/2, monoclonal pAkt antibody (Ser473) and polyclonal Akt antibody were purchased from Cell Signaling Technology/New England Biolabs. Monoclonal Ki67 antibody was obtained from Santa Cruz Biotechnology.

### Preparation of protein-peptide conjugates

500 µg of GRGDSPK or GRADSPK peptides (from BACHEM) in 500 µl PBS were mixed with 500 µg BSA (fatty acid-free, in phosphate buffered saline (PBS)). 250 µl of glutaraldehyde (20 mM in Aqua dest.) were added dropwise and allowed to react for 1 h at room temperature. After the addition of 1 M glycine (in PBS; final concentration 10 mM) the reaction mixture was dialyzed versus PBS overnight.

### Cell lines, culture conditions and DNA-profiling

SCLC cell lines GLC-2 [17] and -36 [18] were provided by Prof. Dr. L.F.M.H. de Leij (University of Groningen, The Netherlands) and SCLC-24H [19] by Dr. H. Lahm (Thoraxklinik Heidelberg, Germany). SCLC cell line H146 was purchased from American Type Culture Collection (Rockville, USA) and SCLC cell line H1184 from DSMZ (Braunschweig, Germany). Cell lines were maintained in DMEM, 10% fetal calf serum (FCS) or in DMEM/Ham'sF12, 20% FCS (cell line H1184). For DNA profiling, 15 different short tandem repeat (STR) systems and the gender specific amelogenin locus were amplified from 1 to 5 ng genomic DNA in a multiplex-PCR using the PowerPlex® 16 System (Promega, Madison, WI, USA), dedicated to forensic DNA profiling, according to manufacturer's instructions. PCR products were quality controlled utilizing a micro fluidic ‘DNA 7500’ array on a 2100 Bioanalyzer (Agilent, Böblingen, Germany). If quality control was passed, allele detection and identification was performed by capillary electrophoresis performed on a 310 Genetic Analyzer (Applied Biosystems, Darmstadt, Germany). Data processing and analysis was done using the GeneMapper software (v3.2) (Applied Biosystems). The DNA profiles of the cell lines are shown in Table 1.

**Table 1:** DNA fingerprinting of SCLC cells.

Cell-Line	GLC-2	GLC-36	H1184	H146	SCLC-24H
STR-System					
D3S1358	17	15,16	14	11	15
TH01	6, 9.3	9, 10	6, 9.3	6, 9.3	9.3
D21S11	30, 32	28, 30	32.2	30	29, 31.2
D18S51	15, 18	12	16, 20	15, 17	14, 15
Penta E	7, 10	5, 10	5, 7	12	12, 13
D5S818	12, 13	9, 12	12	12	11, 12
D13S317	14	11	11	11, 12	12
D7S820	9, 12	8, 12	10, 12	9, 10	11
D16S539	12	12	11	11	12
CSF1PO	12, 13	10	10	11, 12	10
Penta D	11, 13	10, 14	12	13	9
vWA	16	17	16, 17	14, 16	17
D8S1179	12	9, 12	10, 11	12, 14	12, 13
TPOX	11	8	8, 9	8, 11	8, 9
FGA	21, 23	23, 25	22	23	22, 23
AM	XY	XX	XY	XX	XY

AM: Amelogenin (XX: female, XY: male)

### RT-PCR analysis

Total cellular RNA was isolated with the RNeasy Mini Kit (Qiagen) as described in the manufacturer's instructions and 1 µg of RNA was reverse transcribed with the Superscript III kit and random hexamer primers (Life Technologies). cDNAs were amplified (35 to 40 PCR cycles) with gene specific primers (Table S1), the PCR products electrophoresed on 1% agarose gels and visualized with ethidium bromide.

### Cell extraction and Western blot analysis

Cell solubilisation in the presence of protease and phosphatase inhibitors and Western blot analyses were carried out as described [20].

### Two-dimensional gel electrophoresis

Cell lysates were prepared with M-PER Mammalian Protein Extraction Reagent (Thermo Scientific) and centrifuged for 10 min at 10000 g. The cleared supernatant was then precipitated with DOC/TCA [21]. The resulting pellet was dissolved in rehydration buffer (8 M Urea, 2 M Thiourea, 2% w/v CHAPS, 0.002% w/v bromphenol blue, 1% Ampholytes, IPG buffer pH 3–11, 20 mM DTT) and 100 µg protein was applied to IPG strips (ReadyStrip 24 cm, 3–10 NL, BioRad). The IEF run was performed in a Protean IEF cell (BioRad) according to manufactures' instructions. The second dimension separation was carried out on a 12% SDS-PAGE gel according to Laemmli (Ettan Daltsix electrophoresis system). Proteins were visualized with Flamingo Fluorescent Gel Stain (BioRad) and visualized on a FLA-7000 Phosphoimager (Fujifilm). Gel overlays and subsequent analyses were performed with the Delta2D Software Package (Decodon).

### Cell adhesion and differentiation assays

ECM proteins (20 to 40 µg/ml in PBS) or polyornithine (PO; 100 µg/ml in PBS) were spotted as 4 µl droplets in Petri dishes (6 cm in diameter, bacterial grade) and incubated for 2 h at room temperature. Dishes were washed once in PBS, cell suspensions (10^6^ cells/ml in DMEM, 1% BSA; 3 ml per dish) were added and incubated for 2 to 8 h. To remove unbound cells, dishes were washed two times with DMEM, 1% BSA and the number of substrate-adhering cells in defined areas was determined microscopically. For the induction of neuronal differentiation, adherent cells were further cultivated in DMEM, 10% FCS in the absence or presence of 20 or 50 nM SSP.

### Cell proliferation and toxicity assays

Cytotoxicity and cell proliferation were analyzed with the “LDH Cytotoxicity Assay Kit” from Roche. To assess cell toxicity, 10000 cells (in 100 µl culture medium) were seeded per well in 96 well plates. 24 h later, cells were exposed to different concentrations of SSP for 24 h. After treatment, LDH activity was determined in the cell culture supernatants. In parallel, cells that had been treated identically were lysed in order to determine the total LDH activity. For the analysis of cell proliferation, 5000 cells (in 100 µl culture medium) were seeded per well in 96 well plates and allowed to adhere overnight. SSP was then added at concentrations of 20 or 50 nM and cells were further cultivated for 24 to 72 h. Cells were lysed and total LDH activity was determined. In some wells, LDH activity was measured in the culture supernatant to evaluate the presence of cytotoxic effects.

### Flow cytometric analysis

Treated and untreated control cells were trypsinized and collected in PBS and stained with AnnexinV to detect cell death and Ki67 to analyze the cell cycle. Flow cytometry was performed on a BD FACSCanto II instrument, and the data were analyzed with FlowJo software. For cell death detection analysis, the probes were incubated with Annexin-V conjugated to FITC (BD Pharmigen) for 20 min in annexin buffer (BD Pharmigen) on ice, then washed and viable cells were revealed by DAPI (Molecular Probes) exclusion.

To analyze the cell cycle status, cells were fixed and permeabilised following BD BrdU Flow kit manufacturer's instructions, incubated with anti-human Ki67 FITC conjugated antibody (BD Pharmigen) and counterstained with DAPI. Briefly, cells were first resuspended in BD Cytofix/Cytoperm Buffer for 15 to 30 min. Then, probes were washed with BD Perm/Wash Buffer and incubated with BD Cytoperm Plus Buffer 10 min on ice. After washing with BD Perm/Wash Buffer the cells were fixed again for 5 minutes in BD Cytofix/Cytoperm Buffer. After washing away the fixative solution, the probes were stained on ice for one hour with anti-human Ki67-FITC antibody diluted in Perm/Wash Buffer. After washing out the excess of antibody with Perm/Wash Buffer, the cells were resuspended in Perm/Wash Buffer and counterstained with DAPI to exclude debris and necrotic cells.

### Chromosomal analysis

Cells were arrested in metaphase by treatment with colcemid for 4 h. Cells were then subsequently fixed and repeatedly washed in ice-cold methanol: acetic acid (3∶1), applied onto slides and embedded in DAPI mount (Anti-fade Gold, Invitrogen). Chromosome numbers in metaphase nuclei were assessed using a fluorescence microscope. Statistical significance values were calculated in Prism 5.0 software (GraphPad) using Student's t-test with Welch's correction.

### Video time lapse analysis

4000 SCLC cells in 500 µl culture medium were applied to 24-well plates precoated with PO or ECM constituents and allowed to adhere overnight. One hour prior to time-lapse analysis SSP was added to a final concentration of 50 nM and analysis was carried out as described [20]. Briefly, plates were transferred to a heated (37°C), gassed (5% CO_2_/air) and humidified chamber fitted onto an inverted microscope (Leica DM IRE2 HC Fluo) with a motorized cross-stage. Images were recorded every 10 min for 24 h.

## Results

### SSP mediates neurite-like process formation in SCLC cells in an ECM-dependent manner

**Figure 1:**
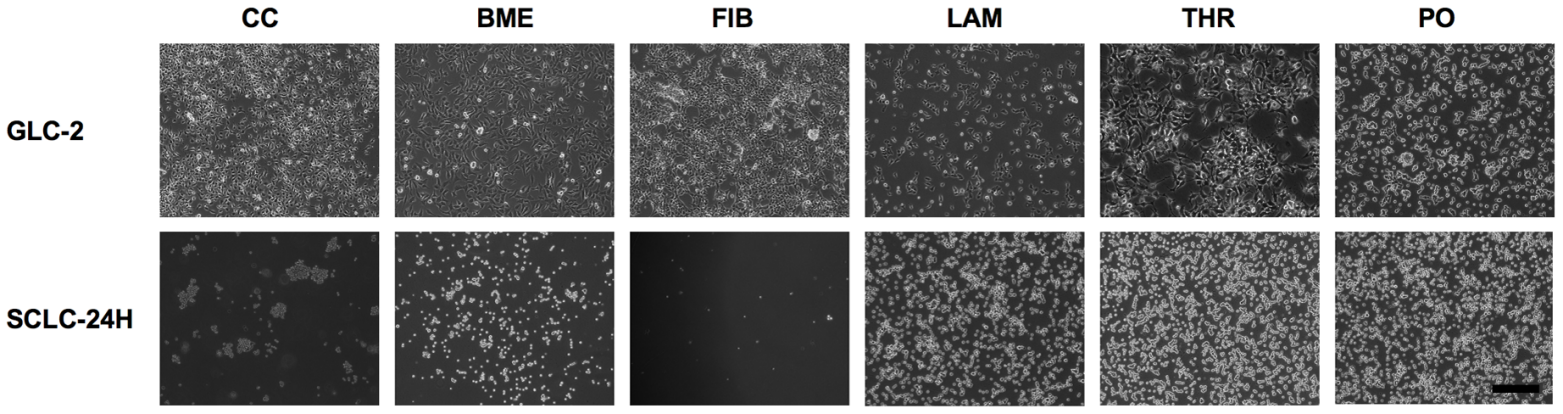
Adhesion of SCLC cells to ECM constituents. GLC-2 or SCLC-24H cells that had been cultivated on cell culture (CC) plastic in complete culture medium were allowed to adhere to BME, FIB, LAM or PO surfaces in DMEM, 1%BSA for 8h. Micrographs were taken after unbound cells had been removed. Bar: 200 µm.

Under normal culture conditions, H146 and SCLC-24H cells grow mainly as aggregates in suspension, GLC-2 as flattened adherent cells and GLC-36 as roundish non-flattened adherent cells. H1148 cell line consists out of two populations: about 80% of the cells grow as flattened adherent cells and the remaining 20% grow as small cell aggregates in suspension. To analyze if SCLC cells differentiate into a neuron-like phenotype, SSP treatment was used as experimental paradigm. Because ECM constituents are known to mediate neurite formation, we first examined the adhesion of SCLC cells to BME, FIB, LAM, THR or PO in a semiquantitative cell adhesion assay (Fig. 1; Table 2). Dependent on the cell line, the adhesion characteristics varied. All cell lines adhered to THR and PO, but only four to BME and FIB and only three to LAM (Table 2). With the exception of GLC-36 cells, SCLC cells showed a flattened morphology on ECM substrata and for GLC-2 cells even small processes were observed (Fig. 1). On PO, a flattened morphology was less pronounced (for GLC-2, H146 and H1184 cells) or absent (for GLC-36 and SCLC-24H cells; Fig. 1).

**Table 2:** Adhesion of SCLC cells to ECM constituents.

ECM component	BME	FIB	LAM	THR	PO
Cell line					
GLC-2	++	+++	+++	+++	+++
GLC-36	+++	+++	++	++++	+++
H146	+	+	++	++	++
H1184	+++	+++	+++	++	++
SCLC-24H	+	-	+++	+++	+++

ECM constituents or PO were immobilized on plastic surfaces and offered as substrates. Protein concentrations used for coating were 20 µg/ml for BME, FIB and LAM, 40 µg/ml for THR and 50 µg/ml for PO. On a total area of 500 µm^2^ the subarea covered with adherent cells was estimated. Values are given semiquantitatively: "−" less then 10, "+" 11–30, "++" 31–60; "+++," 61–80 and "++++" 80–100% of the total area were covered with adherent cells.

**Figure 2:**
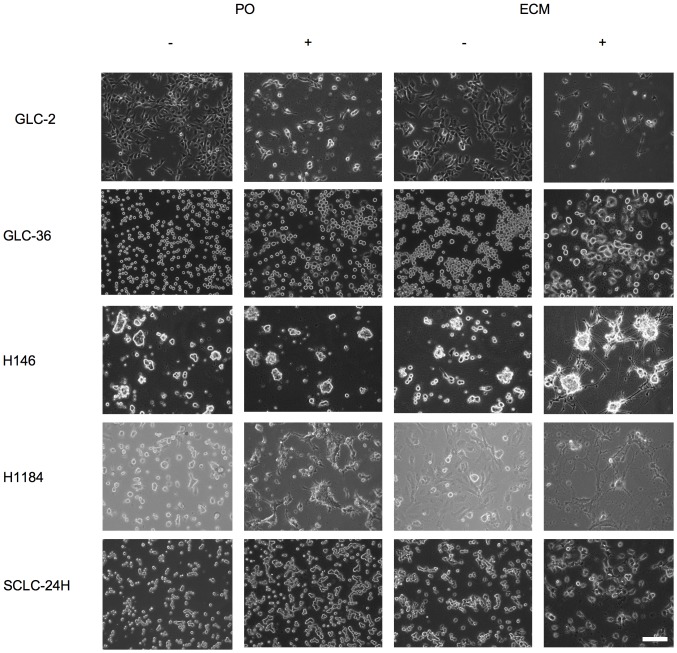
SSP-mediated neurite-like outgrowth of SCLC cells on ECM constituents. Cells were seeded on PO- or ECM-coated Petri dishes (a FIB substrate was used, with the exception of SCLC-24H cells, where LAM was used as a substratum) and cultured for 24 h in the absence (−) or the presence (+) of 20 nM SSP. Bar: 100 µm.

**Figure 3:**
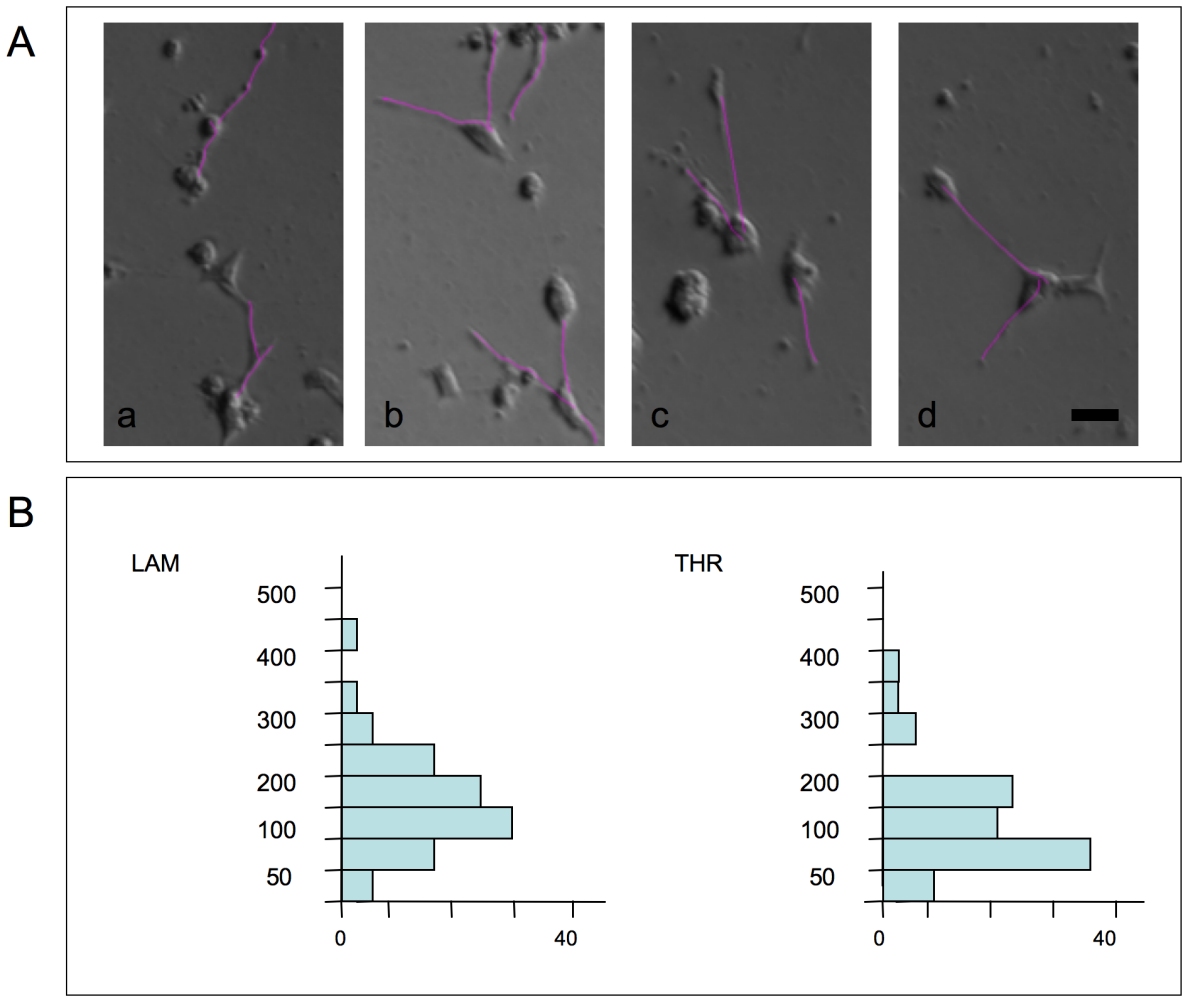
Video time laps analysis of SSP-mediated neurite-like outgrowth of SCLC cells on ECM constituents. 12h-lasting time laps analyses of GLC-2 cells cultured at low density on LAM or THR surfaces in the presence of 50 nM SSP. Tracing of neurite-like extensions was accomplished with Image J software. 80 or 86 cells were analyzed for LAM or THR substrates, respectively. **A:** Selected micrographs of process-bearing GLC-2 cells on LAM (a, b) or THR substrata (c, d). Purple lines indicate traced neurite-like extensions. Bar in d: 20 µm. **B:** Graphs show the sum of total neurite-like process lengths from individual cells for the 12 h period. Distances plotted on the y-axis are split into 50 µm bins and the number of the sums of total neurite-like process lengths (per individual cell) for each category (in percent) is given on the x-axis.

When ECM-adherent SCLC cells were treated with 20 or 50 nM SSP, long neurite-like processes extended from GLC-2, H1184, H146 and SCLC-24H but not from GLC-36 cells (Fig. 2, Table 3). In contrast, cells cultivated on PO showed a pronounced flattening and even small processes emerged (especially for a minor fraction of GLC-2 cells), but neurite-like extensions were never seen, with the exception of H1184 cells that extended neurite-like processes already on unmodified cell culture plastic. In addition, a minor fraction of GLC-2 cells extended processes of minor length. SSP-induced process formation took place fairly rapidly: in video time lapse analyses of SSP-treated GLC-2 cells, velocities of neurite-like extension were 13.1 µm/h on LAM and 10.8 µm/h on THR substrata in average, with maximal values of 34.0 µm/h (on LAM) and 30.6 µm/h (on THR; Fig. 3).

**Table 3:** SSP-induced neurite-like process formation of SCLC cells: dependency on ECM components.

ECM component	BME	FIB	LAM	THR	PO
Cell line					
GLC-2	**++++**	**++++**	**++++**	**++++**	**+**
GLC-36	**-**	**-**	**-**	**-**	**-**
H146	**-**	**-**	++ - ++++*)	**-**	**-**
H1184	**++++**	**++++**	**++++**	**++++**	**+++**
SCLC-24H	**-**	**nc**	**+++**	**-**	**-**

Cells were allowed to adhere to the particular substrates for 2 h and then subsequently treated with 50 nM SSP for 24 h. Values are given semiquantitatively: +: up to 25, ++: 26 to 50, +++: 51 to 75, ++++: 76 to 100% of process-bearing cells; -: no process formation; nc: no cell adhesion. *): Because H146 cells strongly tend to aggregate (see Fig. 2), the number of process-bearing cells could only roughly be estimated.

### Cell adhesion to RGD-containing peptides is sufficient to induce SSP-mediated neurite-like process formation

**Figure 4:**
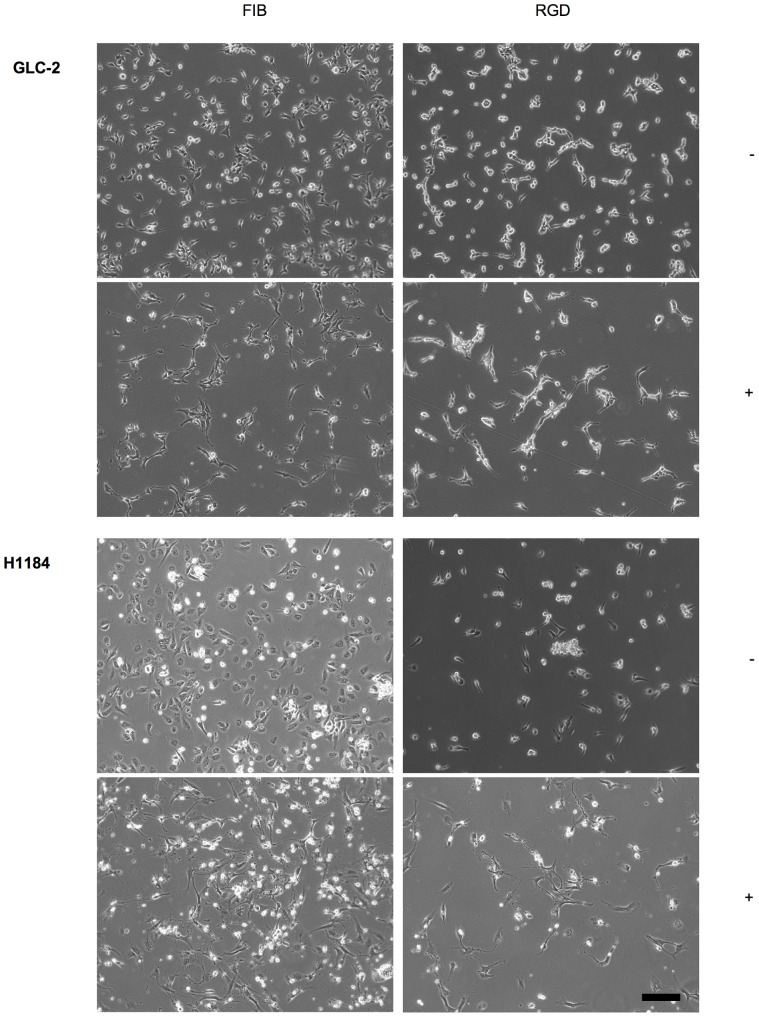
SSP-mediated neurite-like outgrowth of SCLC cells on RGDS-peptides. GLC-2 or H1884 cells adherent to FIB or GRGDSPK-BSA (RGD) substrata were cultivated for 24 h in the absence (−) or presence (+) of 50 nM SSP. Bar: 40 µm.

As the RGD motif is present in many ECM proteins and supports cell adhesion in most of them [22], we investigated the potential of SCLC cells to adhere to and to extend neurite-like processes on RGD-containing peptides. All SCLC cells (except of SCLC-24H cells) adhered to an immobilized GRGDSPK-BSA conjugate, although to a lesser extent when compared with the adhesion to FIB. No adhesion was observed to the control conjugate GRADSP-BSA, to an RGDS-BSA conjugate or to mock-treated BSA (Fig. 4; Table 4). The inability of cells to adhere to the RGDS-BSA conjugate is most likely due to molecular changes of the peptide during the coupling procedure that are incompatible with RGD-integrin recognition. Adhesion efficiency to the GRGDSPK-BSA-conjugate was reduced by about 50% when compared to maximal adhesion to intact ECM molecules. Apart from GLC-36 cells, in the presence of SSP all adherent cells generated neurite-like processes on the GRGDSPK-BSA conjugate that were morphologically comparable to those generated on intact ECM molecules, suggesting that integrin-mediated adhesion to RGDS sequences is sufficient to induce SSP-mediated neurite-like process formation in SCLC cells.

**Table 4:** Adhesion and neurite-like process formation of SCLC cells on RGD peptides.

Cell line	Adhesion			Neurite-like extension		
	GRGDSPK	GRADSPK	FIB (LAM)	GRGDSPK	GRADSPK	FIB (LAM)
GLC-2	++	−/+	+++	+	-	+
GLC-36	++	-	++++	-	na	-
H1184	++	+	+++	+	-	+
SCLC-24H	-	-	++	na	na	+

Cells were allowed to adhere or FIB, LAM (SCLC-24H only) substrata or to immobilized GRGDSPK- or GRADSPK-BSA-conjugates and then treated with 50 nM SSP (for details, see text). na; not applicable.

### SSP-induced neurite-like process formation is reversible

**Figure 5:**
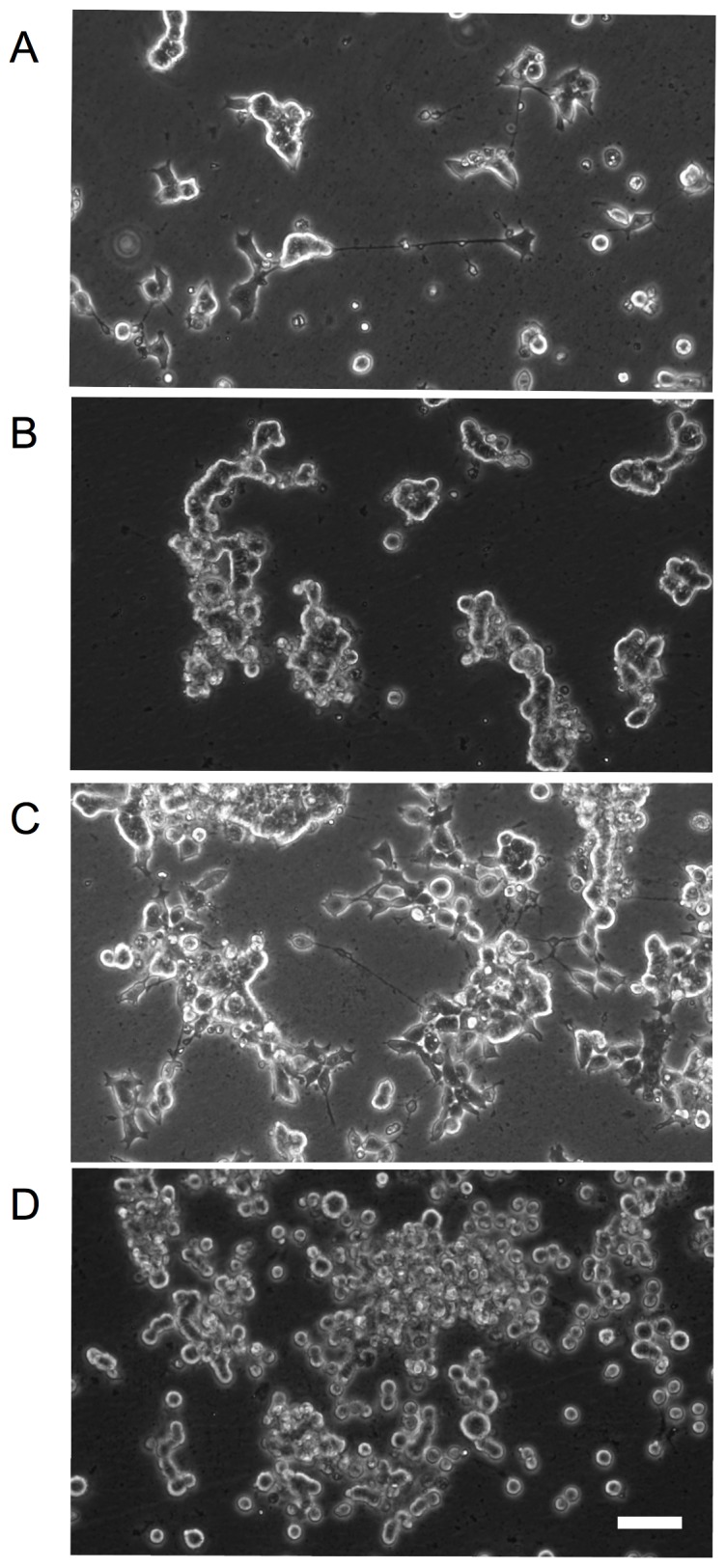
SSP-mediated neurite-like outgrowth of SCLC is reversible. SCLC-24H cells were cultured on a LAM surface in 24 h intervals either in the presence (+) or absence (−) of 50 nM SSP. Culture periods shown are (**A**) 24 h (+), (**B**) 27 h (-; 3 h after first SSP removal); (**C**) 5 days (+) and (**D**) six days (−). Bar: 30 µm.

When SCLC-24H cells that had been treated with 50 nM SSP for one day were further cultured in the absence of the drug, retraction of processes became visible within two to three hours (Fig. 5). New addition of SSP 24 h later again led to neurite-like process extension. This alternation between process formation and retraction could be induced for at least three times (Fig. 5). Comparable results were obtained with GLC-2, H-146 and H1184 cells. Thus, SSP-induced neurite-like process formation needs a continuous presence of the drug.

### SSP-induced neurite-like process formation is independent of canonical signalling pathway activation

**Figure 6:**
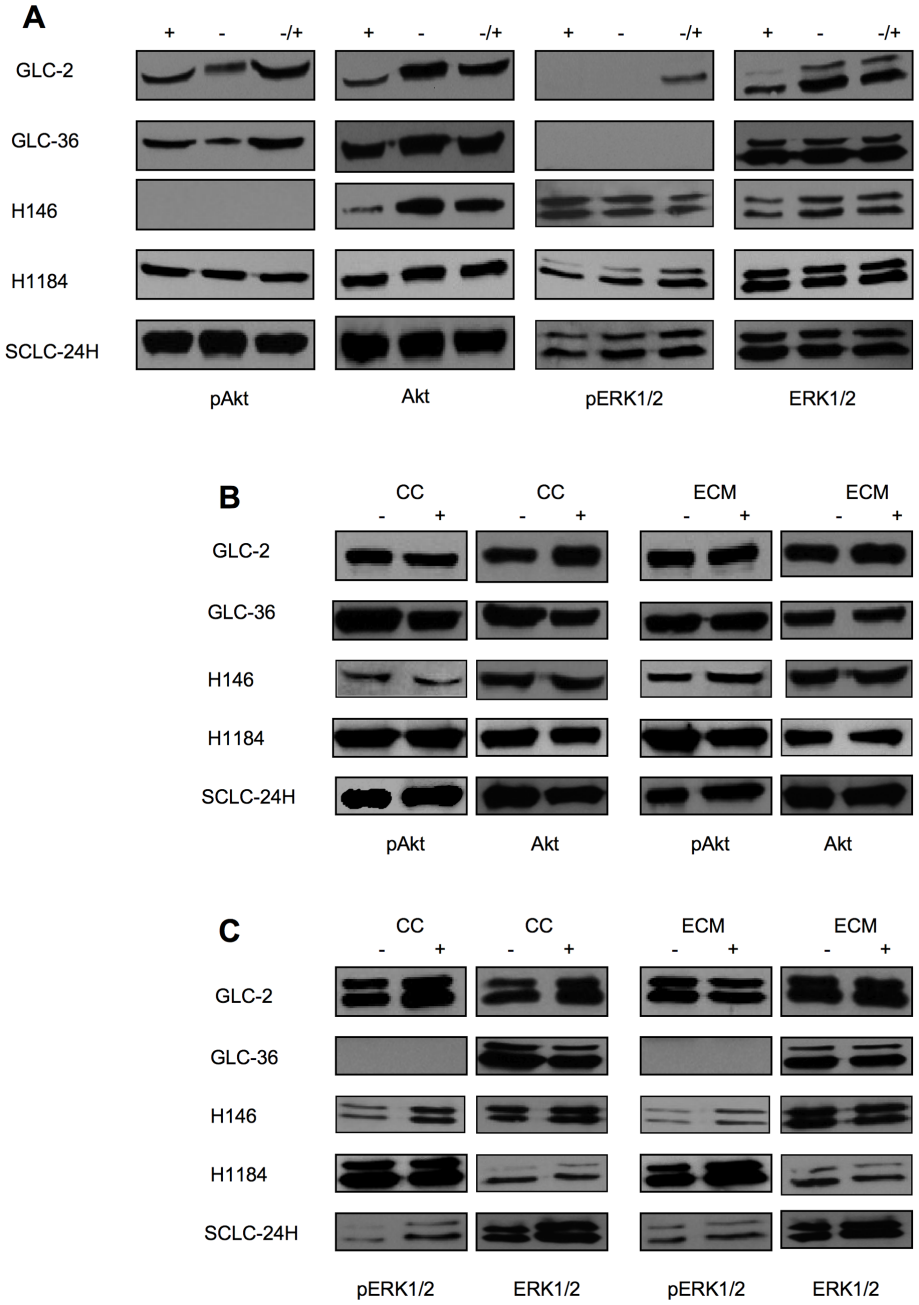
Western blot analysis of Akt and ERK1/2 activation in SCLC cells. (**A**) SCLC cells were cultured for 24 h in the presence (+) or in the absence of serum (−) or in the presence of serum only during the last 10 min of the 24 h culture period (−/+). (**B, C**) SCLC cells were cultured for 24 h in the absence (−) or presence (+) of 50 nM SSP either on cell culture plastic (CC) or on ECM constituents, whereby FIB was used for all cell lines with the exception of SCLC-24H cells, that were cultured on a LAM substrate. Cell lysates were analyzed for the presence of total or phosphorylated (p) Akt (**B**) or ERK1/2 (**C**) proteins. Equal amounts of protein (20 µg) were loaded per lane.

To evaluate if the canonical Ras/MAPK and PI3/Akt pathways are constitutively activated in SCLC cells, cells were cultured for 24 h either in the presence or absence of serum, or for 24 h in the absence of serum and finally for 20 min in the presence of serum and then analyzed for the expression of phosphorylated ERK1/2 (pERK1/2) or Akt (pAkt) (Fig. 6A). Constitutive expression of pAkt by all cell lines was not altered by different serum-containing culture conditions. With the exception of GLC-36 cells, also all cells constitutively expressed pERK1/2. The activation level of Akt und ERK1/2 showed no considerable variation, when cells were cultivated on immobilized ECM proteins (Fig. 6B, C). Upon SSP application, slight alterations in the activation pattern of Akt and ERK1/2 emerged, i.e. an increased expression of pAkt in SCLC-24H (Fig. 6B) and of pERK1/2 in H-146 and SCLC-24H cells (Fig. 6C). These alterations were independent of the ECM substrates used.

**Figure 7:**
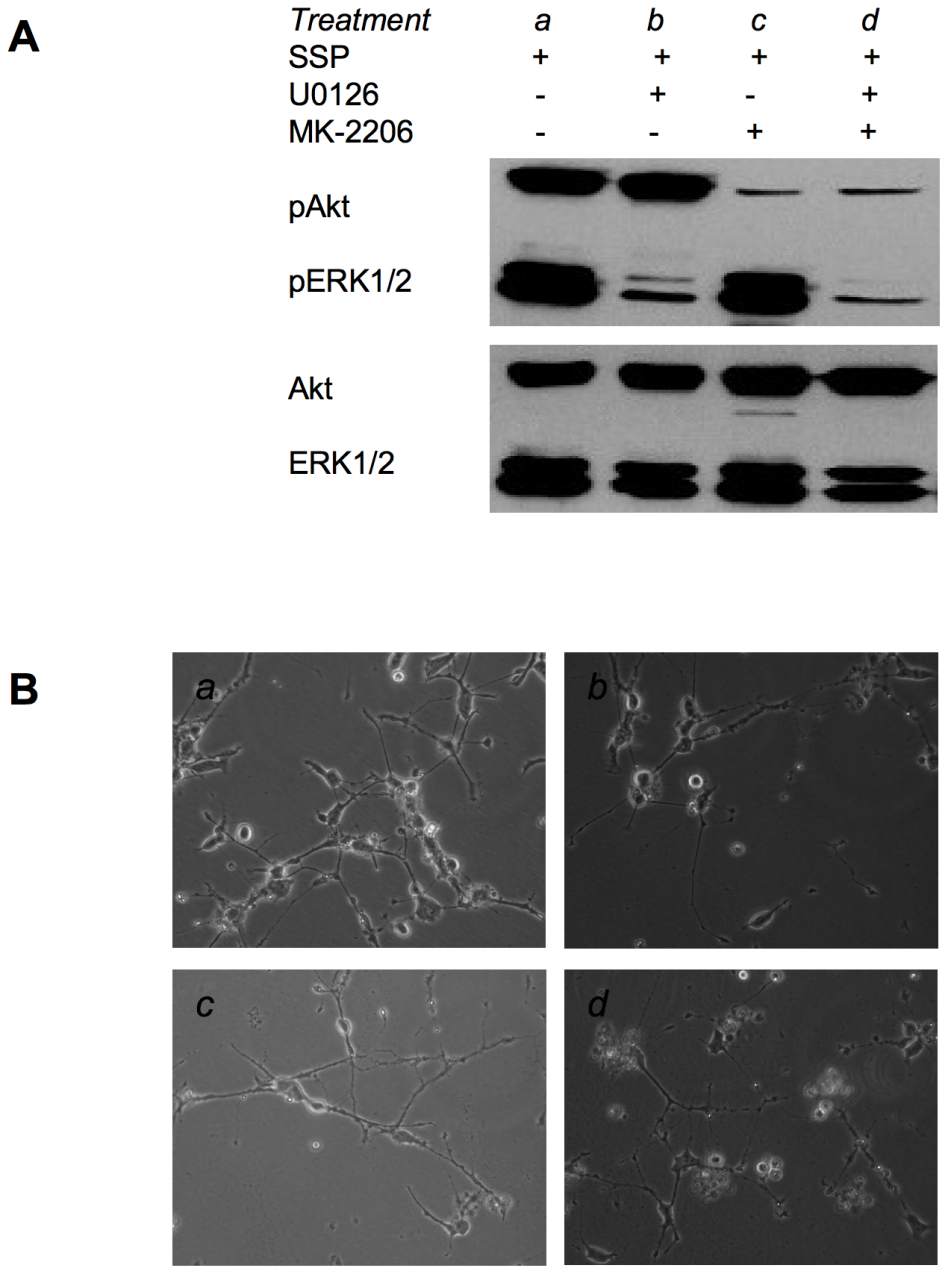
Pharmacological inhibition of Akt and ERK1/2 activation in SCLC-24H cells. (**A**) Western blot analysis of cell lysates from SCLC-24H cells that had been cultured for 48 h on a LAM substrate in the presence (+) or absence (−) of 10 µM U0126 or MK-2206 and in the presence (+) of 20 nM SSP for the final 24 h of the culture period. Cell lysates were analyzed for the presence of total or phosphorylated (p) Akt or ERK1/2 proteins. (**B**) Micrographs of SCLC-24H cells that had been cultured under various conditions as described in (A) and small letters refer to the respective treatments. Equal amounts of protein (20 µg) were loaded per lane.

When SCLC-24H cells were treated for 24 h with 10 µM U0126 or 10 µM MK-2206 the activation of Erk1/2 or Akt was significantly inhibited (Fig. 7A). Under these conditions, however, SSP-induced process formation still took place, indicating an independence of the outgrowth of neurite-like extensions on the activation of these pathways (Fig. 7B).

### SSP does not influence the expression pattern of selected neuroendocrine genes

To analyze if SSP treatment leads to changes in the expression pattern of neuroendocrine-specific genes RT-PCR analyses were performed (Table 5). Most of the SCLC cell lines used in this study expressed a high number of neuroendocrine marker molecules, such as ASH-1, βIII-tubulin, neuron-specific enolase or synaptophysin already under normal culture conditions (Table 5). CD-9, which is predominantly expressed by relapsed SCLC tumors [23], was detected in four out of five cell lines. Galectin-1, whose expression in SCLC in-situ correlates with the proliferation activity of tumor cells [24], was expressed by all cell lines. Surprisingly, galectin-3, that is in-situ expressed by virtually all NSCLC but not SCLC tumors [25], was detectable in all SCLC cell lines. As expected, most (4/5) cell lines express NCAM140 and 180 isoforms, whereas NCAM120 is not expressed [5]. The expression pattern and level of the selected neuroendocrine genes remained constant independent of SSP treatment or ECM substrate used to induce process formation. It is thus likely, that the main molecular machinery necessary for neurite-like outgrowth is already present in untreated SCLC cells.

**Table 5:** RT-PCR analysis of selected gene expression in SCLC cells.

Cell line	GLC-2	GLC-36	H1184	H146	SCLC-24H
Gene					
ASH-1	**+**	**-**	**+**	**+**	**+**
CD-9	**+**	**-**	**+**	**+**	**+**
Galectin-1	**+**	**+**	**+**	**+**	**+**
Galectin-3	**+**	**+**	**+**	**+**	**+**
GAP-43	**+**	**-**	**+**	**-**	**+**
L1-CAM	**+**	**-**	**+**	**+**	**+**
CMYC	**+**	**-**	**+**	**+**	**+**
LMYC	**+**	**-**	**+**	**+**	**-**
NMYC	**+**	**-**	**+**	**+**	**+**
NCAM120	**-**	**-**	**-**	**-**	**-**
NCAM140	**+**	**-**	**+**	**+**	**+**
NCAM180	**+**	**-**	**+**	**+**	**+**
NF-H	**-**	**-**	**+**	**+**	
NF-L	**+**	**-**	**+**	**+**	**+**
NF-M	**+**	**+**	**+**	**+**	**+**
NSE	**+**	**-**	**+**	**+**	**+**
Runx2	**-**	**+**	**+**	**+**	**-**
STP	**+**	**+**	**+**	**+**	**+**
NTRKA	**+**	**-**	**+**	**+**	**+**
NTRKB	**+**	**+**	**+**	**+**	**+**
NTRKC	**+**	**+**	**+**	**+**	**+**
βIII-TB	**+**	**+**	**+**	**+**	**+**

Abbreviations: ASH-1, Achaete-scute complex homologue 1; GAP, growth-associated protein; NCAM, neural cell adhesion molecule; NF, neurofilament; NTRK, neurotrophic tyrosine kinase receptor; NSE, neuron-specific enolase; STP, synaptophysin; TB, tubulin.

### SSP treatment induces alterations in the protein expression pattern

**Figure 8:**
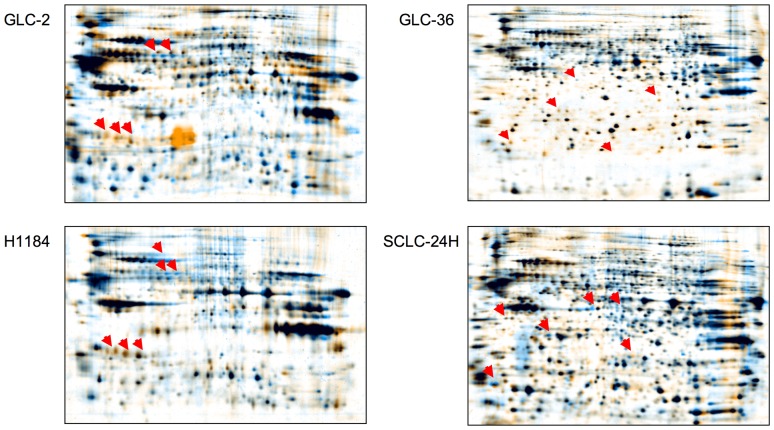
Comparative gel analysis of SCLC cell lysates. Cells were cultivated for 30(blue) or absence (orange) of 20 nM SSP on a FIB (GLC-2, GLC-36, H1184 cells) or a LAM (SCLC-24H cells) substrate. Arrowheads indicate proteins that appear to be differentially expressed in SSP-treated versus untreated cells.

To investigate the possible influence of SSP application on protein expression, an initial set of two-dimensional gels was run with total cell lysates from SCLC cells cultivated for 30 h on ECM substrata in the absence or presence of SSP. As shown in Fig. 8, changes in SSP-treated versus untreated cells could be clearly detected. Further detailed analysis is warranted to identify these proteins and quantify the extent of their regulation, tasks which lie outside the scope of the present study.

### SSP affects the cell death and growth rate of SCLC cells

**Figure 9:**
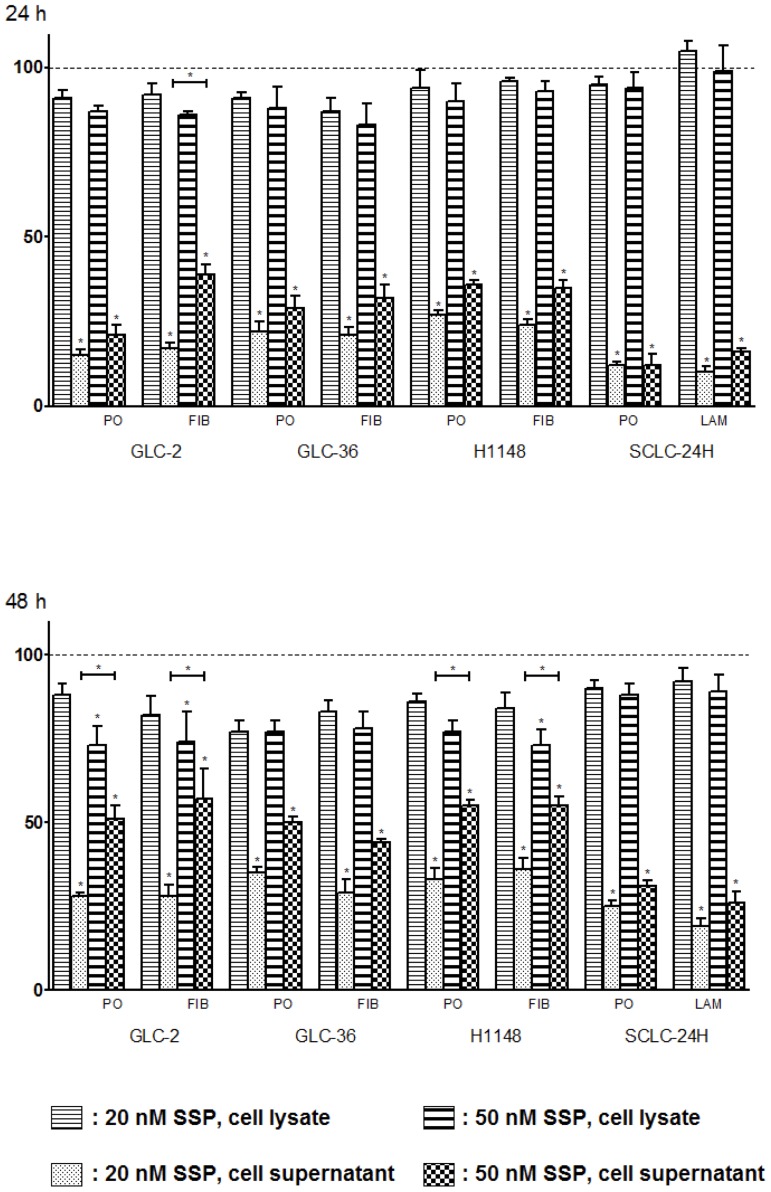
Growth-inhibitory and cytotoxic effects of SSP on SCLC cells as determined by LDH assay. Cells were cultivated for 24 or 48(rate of cytoxicity) or in cell lysates including culture supernatants (rate of cell growth) are shown. The data are means + SD from three to five experiments. One-way ANOVA and the post-hoc Tukey's multiple comparison test were applied using a statistical software program (GraphPad Software, San Diego, CA, USA). Statistically significant differences (*: p<0.05) in comparison to untreated cells are marked with individual asterisks, and between groups treated with 20 or 50 nM SSP with additional bars.

To address the question whether SSP affects the death and/or growth rate of SCLC cells we performed LDH assays with cells that had been either cultured on ECM or on PO substrata, i.e. under conditions when neurite-like process formation takes place or not. Fig. 9 shows that the cytotoxic effect of SSP varied considerably in the different cell lines, but became more pronounced after a culture period of 48 h and with an increasing concentration of SSP. SCLC-24H cells were almost resistant against SSP treatment. For the other cell lines, SSP treatment led up to 30% cytotoxicity after 24 h and to about 50% after 48 h. No obvious differences were observed in cells that had been cultured on PO or on ECM substrata. Increased cytotoxicity was not accompanied by a significant decrease in cell number (determined via the total LDH activity in cell lysates; Fig. 9), most likely indicating an increased cell damage and/or leakiness that still allows a considerable degree of cell proliferation. It has to be mentioned that the presence of 20 or 50 nM SSP for longer than 72 h led to complete cell death in all cell lines. Thus, it is likely that in SCLC cells (i) the action of SSP is mainly cytotoxic but not cytostatic and (ii) that cytoxicity and neurite-like process formation are not interconnected with each other.

### SSP induces multiple alterations during cell cycle progression

**Figure 10:**
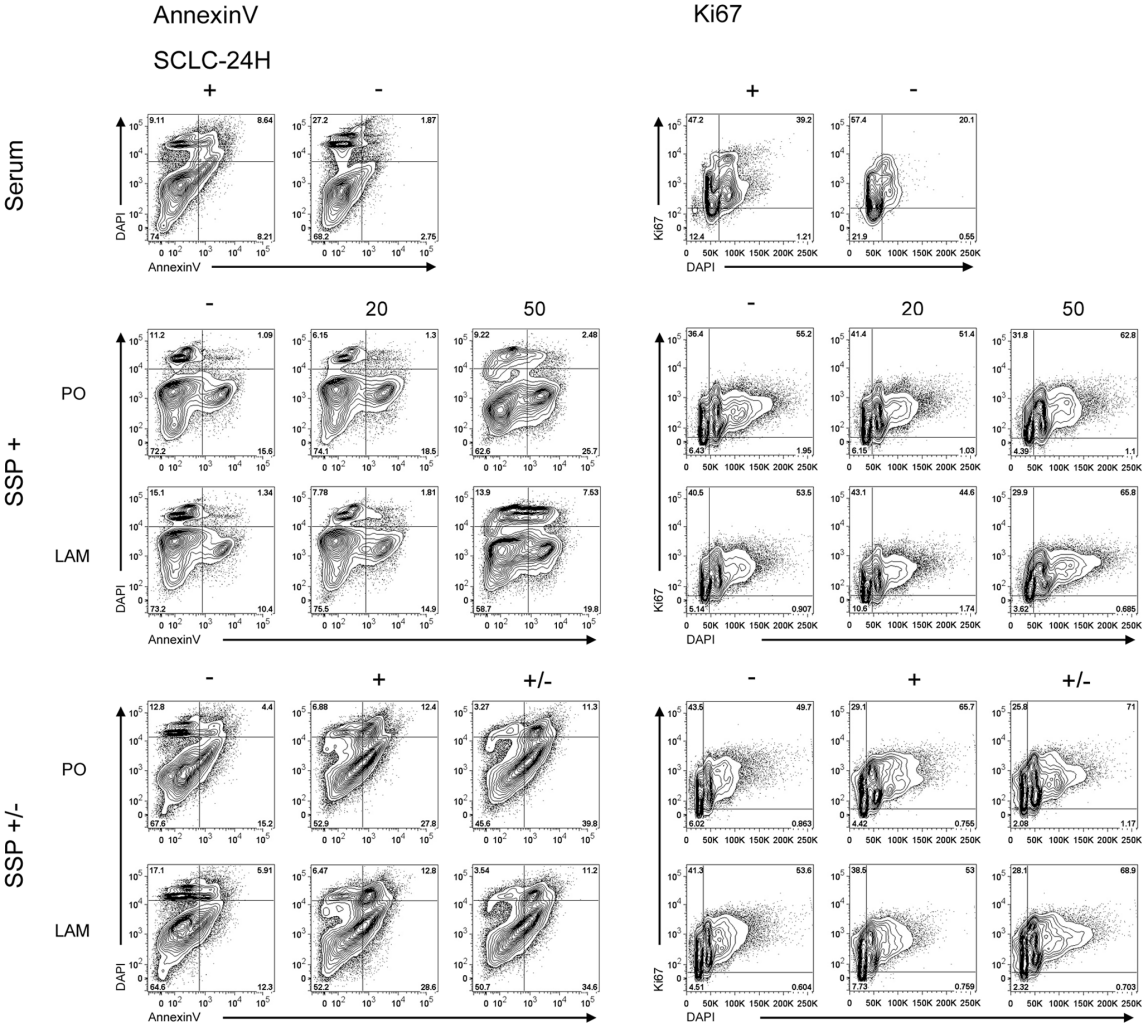
Cell cycle analysis of SCLC-24H cells. Cells were either cultured in the absence or presence of serum (Serum); treated for 24 h without (−) or with different SSP concentrations (20, 50 nM) (SSP+); or treated without SSP for 48 h (−) or with 50 nM SSP for 24 h (SSP +/−) and then analyzed immediately (+) or cultured for further 24 h in the absence of the drug (+/−). Representative FACS plots of cell viability (Annexin V; left panels) and cell cycle progression (Ki67; right panels) under different treatment conditions are shown.

**Figure 11:**
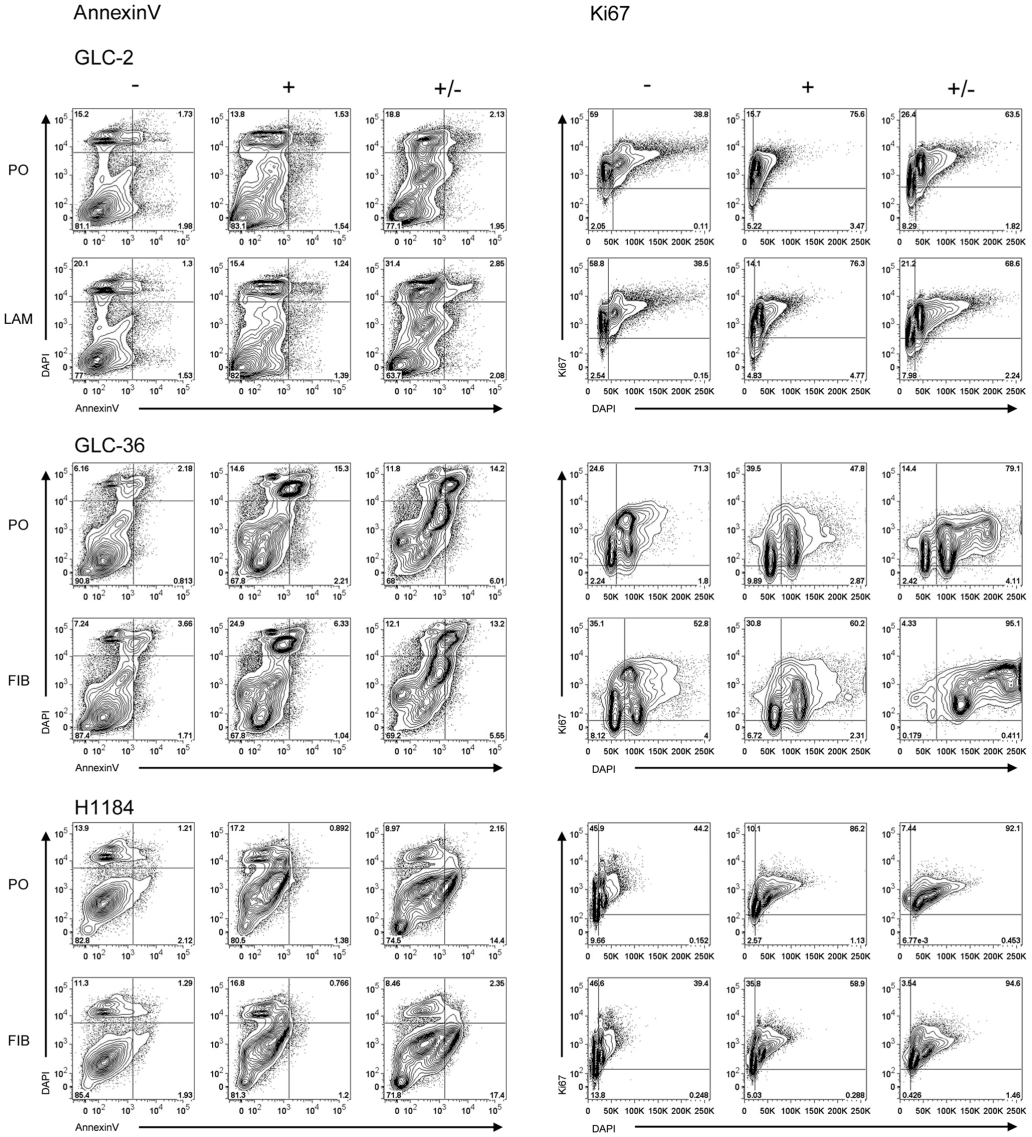
Cell cycle analysis of GLC-2, GLC-36 and H1184 cells. Cells cultured on FIB, LAM or PO were kept untreated (−) or treated with 50 nM SSP for 24 h and analyzed by FACS immediately (+) or cultured for further 24 h in the absence of the drug (+/−). Panels on the left show representative FACS plots of cell viability of the analyzed cell lines (Annexin V). Panels on the right show representative FACS plots of cell cycle progression of the analyzed cell lines (Ki67).

**Figure 12:**
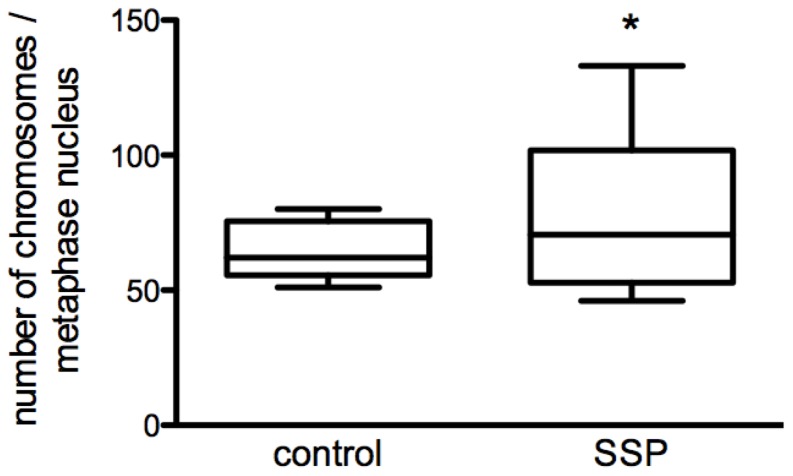
Chromosomal analysis of GLC-36 cells. GLC-36 cells were treated for 24 h without (control) or with 50 nM SSP and then arrested in metaphase by colcemid treatment for further 4 h. Graphs show the mean number of counted chromosomes per metaphase nucleus. N = 17; *: p<0.05.

To analyze more precisely the influence of SSP on the growth characteristics of SCLC cells we performed FACS analyses. When SCLC-24H cells were serum-starved for 24 h, the expected accumulation of cells in G0/G1-phase was observed without obvious signs of increased cell death (Fig. 10). Treatment of SCLC-24H for 24 h with 50 but not with 20 nM SSP, led to an accumulation of cells in the S/G2/M phase with no significant differences in the number of apoptotic as well as of necrotic cells (Fig. 10, Table S2). Thus, in further experiments cells were cultured for one day in the presence of 50 nM SSP and analyzed either immediately or cultured for further 24 h in the absence of the drug. Under these conditions, SCLC-24H cells were mostly arrested in S/G2/M phase, even after a one day cultivation in the absence of SSP (Fig. 10). Independent of the substrate, a significantly reduced cell viability was observed, due to an elevated apoptosis and a moderate necrotic cell death that was still present after the 48 h culture period (Fig. 10, Table S2). H1184 cells were almost completely arrested in S/G2/M phase even 24 h after cultivation in the absence of SSP. Irrespective of the substrate, a small percentage of necrotic cells could be found after SSP incubation that disappeared after removal of the drug (Fig. 11, Table S2). GLC-2 cells were reversibly arrested in S/G2/M phase and moderate necrotic as well as apoptotic cell death appeared that were detectable also after SSP removal in a substrate-independent manner (Fig. 11, Table S2). GLC-36 cells were irreversibly arrested in S/G2/M phase. In this cell line, a moderate increase in apoptotic and necrotic cells was observed that was substrate and treatment independent (Fig. 11, Table S2). As the FACS profile of GLC-36 cells indicated the presence of a polyploidal effect upon SSP treatment, a detailed chromosomal analysis was performed. Most likely due to the perturbed chromosomal structure of GLC-36 cells, initial FISH analyses failed and, thus, the total number of chromosomes had to be determined microscopically. As shown in Fig. 12, the number of chromosomes was indeed significantly increased in cells that were cultivated for 24 h in the presence of 50 nM SSP.

## Discussion

In the present study, we have provided evidence that SCLC cells harbour the potential to develop a neuron-like phenotype. This conversion process - that can be induced by SSP when RGDS-integrin interactions are allowed to take place in parallel - has the following characteristics: (i) it takes place rapidly; i.e. within hours, (ii) it is reversible (also in matter of hours), (iii) it does not require any activation or inactivation of canonical signalling pathways (Ras/MAPK or PI3/Akt), (iv) it does not need neuroendocrine gene neoexpression, and (v) it leads to significant changes in the overall protein expression pattern. Regardless of the presence of ECM proteins, SSP exhibits a cytotoxic as well as a proliferation-inhibiting effect on SCLC cells whereby the latter either leads to a cell cycle arrest in G2/M phase or to polyploidy. The processes that are formed by SCLC cells upon SSP treatment often resemble to neurites, but because (i) we have not analyzed their molecular architecture and (ii) other non-neuronal cells, as for example podocytes [26], can extend processes similar to neurites we think that the neutral term neurite-like is more adequate to describe the processes established by SCLC cells.

### Molecular prerequisites for neurite-like process formation in SCLC cells

SSP is known to interfere with the action of about 90% of all kinases [27]. Therefore, it can serve as an experimental paradigm for a kind of general interference with the action of cellular kinases. In this respect, it is not surprising that SSP influences a variety of cellular activities such as proliferation and differentiation [28], [29] or different types of cell death [30], [31]. It is a self-evident consequence that follow-up studies with more specific compounds are needed, that allow to dissect the widespread repertoire of SSP actions. Indeed, in preliminary experiments we found evidence that treatment of SCLC cells with different SSP analogs can induce less pronounced or more restricted cellular responses.

It is likely, that the molecular prerequisites needed for neurite-like process formation are already expressed by untreated SCLC cells: (i) Process extension occurs in the range of hours, a kinetics that points against the involvement of new gene expression/protein synthesis; (ii) a great number of neuroendocrine marker genes are already expressed in untreated SCLC cells and their expression pattern remains unchanged upon SSP stimulation. Thus, SSP seems to activate an already present conversion pathway but not to induce the expression of its pathway members. But the action of SSP, although necessary, is not sufficient to induce neurite-like extensions and needs the additional presence of RGD-integrin interactions. We are thus confronted with two main questions: (i) which signalling pathways are activated via SSP in SCLC cells, and (ii) which key genes lay the molecular basis for a neuron-like differentiation of these cells?

The signalling pathways that are influenced by SSP are only insufficiently known and can vary dependent on the cell type. In PC12 cells, a c-Jun NH_2_ terminal kinase contributes to SSP-induced neurite outgrowth [32]. The canonical ERK1/2 and PI3/Akt pathways are most likely not involved in SSP-induced neuron-like differentiation because (i) in some cell lines the ERK1/2 pathways is not activated, but SSP still provokes the formation of neurite-like extensions, (ii) pharmacological inhibition of the constitutively activated PI3/Akt pathway in SCLC cells does not prevent neurite-like outgrowth. In addition, neither culturing SCLC cells on ECM substrata (not shown) nor addition of SSP (see Fig. 6) influenced the activation state of Akt or ERK1/2 in SCLC cells.

Integrin-ECM interactions can be of multiple natures in SCLC cells, as they express α2β1, α3β1, α6β1, αvβ1 integrins that bind to collagen and LAM (α2β1, α3β1, α β1), to FIB (α3β1, αvβ1) or to THR (α3β1) [33], [34]. For OH-1 SCLC cells, adhesion to THR is sufficient to induce neurite-like outgrowth and to inhibit proliferation, effects that are enforced in the presence of epidermal growth factor [34]. Murine osteoblast precursor MC3T3-E1 cells are protected from SSP-induced apoptotic cell death when cultivated on a RGDS-, but not on a RGES-substrate via activation of the PI3/Akt pathway [35]. It has been reported that integrin-mediated activation of the PI3/Akt pathway protects SCLC cells from etoposide and radiation induced cell death [36]. Also in osteoblast-like cells, adhesion to an RGDS peptide matrix abolishes SSP-induced apoptosis in a PI3/Akt-dependent manner [35]. In our hands, adhesion to ECM components did not alter the proliferation pattern or induced a chemoprotective effect towards SSP or etoposide in SCLC cells and the reasons for these discrepancies are so far unknown.

One candidate for a key gene for neuronal differentiation of SCLC cells is ASH-1 that is expressed in normal fetal pulmonary endocrine cells, cells that do not develop in HASH-1 knockout mice [37]. In SCLC cells, ASH-1 also positively influences the expression of other neural genes, such as neuron-specific enolase or synaptophysin and modulates tumorgenicity [37], [38]. GLC-36 cells, that do not extend neurites in the presence of SSP, also express the lowest number of neuroendocrine marker molecules, i.e. ASH-1, neuron-specific enolase, neurofilament H-and L or NCAM transcripts could not be detected. Concerning ASH-1 and neurofilament expression, it is notable that in umbilical cord blood cells, which possess stem cell characteristics, SSP induces a neural phenotype accompanied by GFAP, HASH-1 and neurofilament expression [39].

We propose that SSP affects SCLC cells in two distinct ways: it (i) induces neurite-like outgrowth and (ii) leads to cell death. On the molecular level, these two phenomena are most likely independent from each other, as (i) process formation occurs very rapidly and during this initial phase after SSP application no cytotoxic effects are visible and (ii) cytotoxic effects of SSP are also present when cells are cultivated on PO, i.e. under conditions when process formation is abolished.

### SSP-induced cell cycle arrest in SCLC cells

In SCLC cells, SSP treatment led to a cell cycle arrest in the G2/M phase. This is in agreement with previous studies, which demonstrated a cell cycle arrest in G2/M upon SSP treatment in various cancer cell lines [40]. It has also been reported that low SSP concentrations can lead to G1 cell cycle arrest [40], [41]. The SSP analog UCN-01 (7-hydroxySSP) also leads to an accumulation of cells in the G1 phase [42], an effect, which, in irradiated cells, is due to inhibition of the DNA damage checkpoint kinase hChk1 [43]. Thus, it would be of interest to examine which molecular substructures affect the distinct functional properties of SSP [44].

### SSP and SCLC therapy

We have shown that SSP induces intense process formation, but does not lead to cell cycle arrest in SCLC cells. In cervical cancer cells, lower SSP concentrations induce a reversible cell cycle arrest either in G1 or G2/M phase after 24 h, whereas longer incubation times or higher SSP concentrations lead to apoptotic cell death [39]. In neuroblastoma cells, increased neuronal differentiation can be provoked even at high SSP concentrations when apoptotic cell death is inhibited via BCl-X_L_ overexpression. Thus, it can be speculated that SSP induces cell cycle arrest/neuronal differentiation and apoptotic cell death via two distinct mechanisms [45]. In SCLC cells, the anti-cancer effects of SSP we have observed are restricted to cytotoxic effects that lead to apoptotic or necrotic cell death. It remains to be determined if the level of SSP's cytotoxicity towards cancer cells differs from the one of normal cells. Preliminary data of ours indicate that at least normal mesothelial cells tolerated SSP concentrations up to 50 nM without any signs of cell death (R. Probstmeier, unpublished observations). It is also promising that SSP-treated SCLC cells express new sets of proteins. However, their molecular nature needs to be further clarified to evaluate their potential as therapeutic targets.

## Supporting Information

Table S1 **List of primers used for RT-PCR.** Sequences were always chosen to span at least one intron sequence.
(DOC)

Table S2 **Cell cycle analysis of SCLC cells: Quantification of cell viability, apoptotic and necrotic cell death.** SCLC cells were either cultured in the absence (- FCS) or presence of fetal calf serum (+ FCS), or cultured in the presence of serum and treated for 24 h with 20 or 50 nM SSP or for 24 h with 50 nM SSP and then cultured for further 24 h in the absence of the drug (+/−) and analyzed by FACS. Subtables present data obtained for viable (AnnexinV^−^ DAPI^−^; S2-1), apoptotic (AnnexinV^+^; S2-2) or necrotic (AnnexinV^−^, DAPI^+^; S2-3) cell populations. Values are given as percentages (mean values ± standard deviation) of the total number of events analyzed. Statistical difference to control cells cultured in the presence of serum (+ FCS) was assessed by unpaired t-test; *: p≤0.05. FCS: fetal calf serum, FIB: fibronectin; LAM: laminin; PO: polyornithine; SSP: staurosporine.
(DOC)

## References

[1] Travis WD (2010) Advances in neuroendocrine lung tumors. Ann Oncol 21 Suppl 765–71. 10.1093/annonc/mdq380 20943645

[2] Riaz SP, Lüchtenborg M, Coupland VH, Spicer J, Peake MD, et al (2012) Trends in incidence of small cell lung cancer and all lung cancer. Lung Cancer 75: 280–284. 21893364 10.1016/j.lungcan.2011.08.004

[3] Park KS, Liang MC, Raiser DM, Zamponi R, Roach RR, et al (2011) Characterization of the cell of origin for small cell lung cancer. Cell Cycle 10: 2806–2815. 21822053 10.4161/cc.10.16.17012 PMC3219544

[4] Sutherland KD, Proost N, Brouns I, Adriaensen D, Song JY (2011) Cell of origin of small cell lung cancer: inactivation of Trp53 and Rb1 in distinct cell types of adult mouse lung. Cancer Cell 19: 754–764. 21665149 10.1016/j.ccr.2011.04.019

[5] Pedersen N, Mortensen S, Sørensen SB, Pedersen MW, Rieneck K, et al (2003) Transcriptional gene expression profiling of small cell lung cancer cells. Cancer Res 63: 1943–1953. 12702587

[6] Stovold R, Blackhall F, Meredith S, Hou J, Dive C, et al (2012) Biomarkers for small cell lung cancer: neuroendocrine, epithelial and circulating tumour cells. Lung Cancer 76: 263–268. 22177533 10.1016/j.lungcan.2011.11.015

[7] Haddadin S, Perry MC (2011) History of small-cell lung cancer. Clin Lung Cancer 12: 87–93. 21550554 10.1016/j.cllc.2011.03.002

[8] William WN Jr, Glisson BS (2011) Novel strategies for the treatment of small-cell lung carcinoma. Nat Rev Clin Oncol 8: 611–619. 21691321 10.1038/nrclinonc.2011.90

[9] Arriola E, Cañadas I, Arumí M, Rojo F, Rovira A, et al (2008) Genetic changes in small cell lung carcinoma. Clin Transl Oncol 10: 189–197. 18411191 10.1007/s12094-008-0181-1

[10] D'Angelo SP, Pietanza MC (2010) The molecular pathogenesis of small cell lung cancer. Cancer Biol Ther 10: 1–10. 21361067 10.4161/cbt.10.1.12045

[11] Ablain J, de The H (2011) Revisiting the differentiation paradigm in acute promyelocytic leukemia. Blood 117: 5795–5802. 21444912 10.1182/blood-2011-02-329367

[12] Elstner E, Müller C, Koshizuka K, Williamson EA, Park D, et al (1998) Ligands for peroxisome proliferator-activated receptorgamma and retinoic acid receptor inhibit growth and induce apoptosis of human breast cancer cells in vitro and in BNX mice. Proc Natl Acad Sci USA 95: 8806–8811. 9671760 10.1073/pnas.95.15.8806 PMC21158

[13] Azzi S, Bruno S, Giron-Michel J, Clay D, Devocelle A, et al (2011) Differentiation therapy: targeting human renal cancer stem cells with interleukin 15. J Natl Cancer Inst 103: 1884–1898. 22043039 10.1093/jnci/djr451

[14] Campos B, Wan F, Farhadi M, Ernst A, Zeppernick F, et al (2010) Differentiation therapy exerts antitumor effects on stem-like glioma cells. Clin Cancer Res 16: 2715–2728. 20442299 10.1158/1078-0432.CCR-09-1800

[15] Missale C, Codignola A, Sigala S, Finardi A, Paez-Pereda M, et al (1998) Nerve growth factor abrogates the tumorigenicity of human small cell lung cancer cell lines. Proc Natl Acad Sci USA 95: 5366–5371. 9560282 10.1073/pnas.95.9.5366 PMC20267

[16] Giaccone G, Broers J, Jensen S, Fridman RI, Linnoila R, et al (1992) Increased expression of differentiation markers can accompany laminin-induced attachment of small cell lung cancer cells. Br J Cancer 66: 488–495. 1325826 10.1038/bjc.1992.301 PMC1977936

[17] de Leij L, Postmus PE, Buys CH, Elema JD, Ramaekers F, et al (1985) Characterization of three new variant type cell lines derived from small cell carcinoma of the lung. Cancer Res 45: 6024–6033. 2998591

[18] König K, Meder L, Kröger C, Diehl L, Florin A, et al (2013) Loss of the keratin cytoskeleton is not sufficient to induce epithelial mesenchymal transition in a novel KRAS driven sporadic lung cancer mouse model. PLoS ONE 8(3): e57996 doi:10.1371/journal.pone.0057996 23536778 10.1371/journal.pone.0057996 PMC3594220

[19] Lahm H, André S, Hoeflich A, Fischer JR, Sordat B, et al (2001) Comprehensive galectin fingerprinting in a panel of 61 human tumor cell lines by RT-PCR and its implications for diagnostic and therapeutic procedures. J Cancer Res Clin Oncol 127: 375–386. 11414198 10.1007/s004320000207 PMC12164915

[20] Glassmann A, Reichmann K, Scheffler B, Glas M, Veit N, et al (2011) Pharmacological targeting of the constitutively activated MEK/MAPK-dependent signaling pathway in glioma cells inhibits cell proliferation and migration. Int J Oncol 39: 1567–1575. 21850371 10.3892/ijo.2011.1165

[21] Peterson GL (1983) Determination of total protein. Meth Enzymol 91: 95–121. 6855607 10.1016/s0076-6879(83)91014-5

[22] Ruoslahti E (1996) RGD and other recognition sequences for integrins. Annu Rev Cell Dev Biol 12: 697–715. 8970741 10.1146/annurev.cellbio.12.1.697

[23] Kohmo S, Kijima T, Otani Y, Mori M, Minami T, et al (2010) Cell surface tetraspanin CD9 mediates chemoresistance in small cell lung cancer. Cancer Res 70: 8025–8035. 20940407 10.1158/0008-5472.CAN-10-0996

[24] Gabius HJ, André S, Gunsenhäuser I, Kaltner H, Kayser G, et al (2002) Association of galectin-1- but not galectin-3-dependent parameters with proliferation activity in human neuroblastomas and small cell lung carcinomas. Anticancer Res 22: 405–410. 12017323

[25] Buttery R, Monaghan H, Salter DM, Sethi T (2004) Galectin-3: differential expression between small-cell and non-small-cell lung cancer. Histopathology 44: 339–344. 15049899 10.1111/j.1365-2559.2004.01815.x

[26] Kobayashi N (2002) Mechanism of the process formation; podocytes vs. neurons. Microsc Res Tech 57: 217–223. 12012387 10.1002/jemt.10077

[27] Karaman MW, Herrgard S, Treiber DK, Gallant P, Atteridge CE, et al (2008) A quantitative analysis of kinase inhibitor selectivity. Nat Biotechnol 26: 127–132. 18183025 10.1038/nbt1358

[28] Peng HY, Liao HF (2011) Staurosporine induces megakaryocytic differentiation through the upregulation of JAK/Stat3 signaling pathway. Ann Hematol 90: 1017–1029. 21331591 10.1007/s00277-011-1186-3

[29] Schumacher A, Arnhold S, Addicks K, Doerfler W (2003) Staurosporine is a potent activator of neuronal, glial, and “CNS stem cell-like” neurosphere differentiation in murine embryonic stem cells. Mol Cell Neurosci 23: 669–680. 12932446 10.1016/s1044-7431(03)00170-2

[30] Deshmukh M, Johnson EM (2000) Staurosporine-induced neuronal death: multiple mechanisms and methodological implications. Cell Death Differ 7: 250–261. 10745270 10.1038/sj.cdd.4400641

[31] Dunai ZA, Imre G, Barna G, Korcsmaros T, Petak I, et al (2012) Staurosporine Induces Necroptotic Cell Death under Caspase-Compromised Conditions in U937 Cells. PLoS ONE 7(7): e41945 doi:10.1371/journal.pone.0041945 22860037 10.1371/journal.pone.0041945 PMC3409216

[32] Yao R, Yoshihara M, Osada H (1997) Specific activation of a c-Jun NH2-terminal kinase isoform and induction of neurite outgrowth in PC-12 cells by staurosporine. J Biol Chem 272: 18261–18266. 9218464 10.1074/jbc.272.29.18261

[33] Hodkinson PS, Mackinnon AC, Sethi T (2007) Extracellular matrix regulation of drug resistance in small-cell lung cancer. Int J Radiat Biol 83: 733–741. 17852559 10.1080/09553000701570204

[34] Guo N, Templeton NS, Al-Barazi H, Cashel JA, Sipes JM, et al (2000) Thrombospondin-1 promotes alpha3beta1 integrin-mediated adhesion and neurite-like outgrowth and inhibits proliferation of small cell lung carcinoma cells. Cancer Res 60: 457–466. 10667601

[35] Grigoriou V, Shapiro IM, Cavalcanti-Adam EA, Composto RJ, Ducheyne P, et al (2005) Apoptosis and survival of osteoblast-like cells are regulated by surface attachment. J Biol Chem 280: 1733–1739. 15522882 10.1074/jbc.M402550200

[36] Hodkinson PS, Elliott T, Wong WS, Rintoul RC, Mackinnon AC, et al (2006) ECM overrides DNA damage-induced cell cycle arrest and apoptosis in small-cell lung cancer cells through beta1 integrin-dependent activation of PI3-kinase. Cell Death Differ 13: 1776–1788. 16410797 10.1038/sj.cdd.4401849

[37] Borges M, Linnoila RI, van de Velde HJ, Chen H, Nelkin BD, et al (1997) An achaete-scute homologue essential for neuroendocrine differentiation in the lung. Nature 386: 852–855. 9126746 10.1038/386852a0

[38] Jiang T, Collins BJ, Jin N, Watkins DN, Brock MV (2009) Achaete-scute complex homologue 1 regulates tumor-initiating capacity in human small cell lung cancer. Cancer Res 69: 845–854. 19176379 10.1158/0008-5472.CAN-08-2762 PMC2919317

[39] Faghihi F, Mehranjani MS, Mehrjerdi NZ, Baharvand H (2008) Effect of staurosporine on neural differentiation of CD133^+^ umbilical cord blood cells. Yakhthe Med J 10: 33–40.

[40] Bernard B, Fest T, Prétet JL, Mougin C (2001) Staurosporine-induced apoptosis of HPV positive and negative human cervical cancer cells from different points in the cell cycle. Cell Death Differ 8: 234–244. 11319606 10.1038/sj.cdd.4400796

[41] McGahren-Murray M, Terry NH, Keyomarsi K (2006) The differential staurosporine-mediated G1 arrest in normal versus tumor cells is dependent on the retinoblastoma protein. Cancer Res 66: 9744–9753. 17018634 10.1158/0008-5472.CAN-06-1809

[42] Senderowicz AM (2003) Small-molecule cyclin-dependent kinase modulators. Oncogene 22: 6609–6620. 14528286 10.1038/sj.onc.1206954

[43] Busby EC, Leistritz DF, Abraham RT, Karnitz LM, Sarkaria JN (2000) The radiosensitizing agent 7-hydroxystaurosporine (UCN-01) inhibits the DNA damage checkpoint kinase hChk1. Cancer Res 60: 2108–2112. 10786669

[44] Thompson AF, Levin LA (2010) Neuronal differentiation by analogs of staurosporine. Neurochem Int 56: 554–560. 20043966 10.1016/j.neuint.2009.12.018 PMC2831141

[45] Yuste VJ, Sánchez-López I, Solé C, Encinas M, Bayascas JR, et al (2002) The prevention of the staurosporine-induced apoptosis by Bcl-X(L), but not by Bcl-2 or caspase inhibitors, allows the extensive differentiation of human neuroblastoma cells. J Neurochem 80: 126–139. 11796751 10.1046/j.0022-3042.2001.00695.x

