# Supplementary material for: Staurosporine and Extracellular Matrix Proteins Mediate the Conversion of Small Cell Lung Carcinoma Cells into a Neuron-Like Phenotype
Source: PLoS One. 2014 Feb 28;9(2):e86910. doi: 10.1371/journal.pone.0086910 (PMC3938400; doi:10.1371/journal.pone.0086910)
Supplement: Table S1 — List of primers used for RT-PCR. Sequences were always chosen to span at least one intron sequence. (DOC) [file pone.0086910.s001.doc]

**Supplemental data Table S1 - List of primers used for RT-PCR**

| **Gene** | **Primer sequences** | **Product size** |
| --- | --- | --- |
| **ASH-1.** Official name: Achaete-scute complex homolog 1 (Drosophila); Official symbol: ASCL1 | up: CATCTCCCCCAACTACTCCA | 366 |
|  | do: CCGTTTTCTGAAAGCCATGT |  |
| **CD9.** Official Name: Homo sapiens CD9 molecule; Official Symbol: CD9 | up: TGCATCTGTATCCAGCGCCA | 799 |
|  | do: CTCAGGGATGTAAGCTGACT |  |
| **Galectin-1.** Official name: lectin, galactoside-binding, soluble, 1 Official symbol: LGALS3 | up: AACCTGGAGAGTGCCTTCGA | 321 |
|  | do: GTA GTTGATGGCCTCCAGGT |  |
| **Galectin-3.** Official name: lectin, galactoside-binding, soluble, 3; Official symbol: LGALS1 | up: GGCCACTGATTGTGCCTTAT | 302 |
|  | do: TGCAACCTTGAAGTGGTCAG |  |
| **GAP-43 variant 1 & 2.** Official name: growth associated protein 43; Official symbol: GAP43 | up: CGATGAGCAATAGCTGTGGA | variant 1: 704 |
|  | do: TGCGGCCTTATGAGCTTTAT | variant 2: 309 |
| **L1CAM.** Official name: Homo sapiens L1 cell adhesion molecule (L1CAM), transcript variant 3, mRNA; Official Symbol: L1CAM | up: GAGTAGGGCACAGCTCTTGG | 290 |
|  | do: CGGGGCCATATTTGTTTATG |  |
| **L-Myc.** Official Name: Homo sapiens v-myc myelocytomatosis viral oncogene homolog 1, lung carcinoma derived (avian); Official Symbol: MYCL1 | up: GCATGTGCGTGTGTGCTG | 365 |
|  | do: GCGTATGATGGAGGCGTAGT |  |
| **N-Myc.** Official Name: Homo sapiens v-myc myelocytomatosis viral related oncogene, neuroblastoma derived (avian) mRNA; Official Symbol: MYCN | up: ACACCCTGAGCGATTCAGAT | 399 |
|  | do: GCTCCAGGATGTTGTGGTTT |  |
| **CMYC.** Official Name: Homo sapiens v-myc myelocytomatosis viral oncogene homolog (avian); Official Symbol: MYC | up: AGAGAAGCTGGCCTCCTACC | 398 |
|  | do: CTCTGACCTTTTGCCAGGA |  |
| **NCAM-120.** Official Name: Neural cell adhesion molecule 1; Official Symbol: NCAM1(transcript variant 4) | up: CTAAGCTCGAAGGGCAGATG | 420 |
|  | do: TCACTGCAGAGAAAAGCAATG |  |
| **NCAM-140.** Official Name: Neural cell adhesion molecule 1; Official Symbol: NCAM1(transcript variant 1) | up: ACGGCAGCCCCACCTCAG | 434 |
|  | do: ATCATGCTTTGCTCTCGTTC |  |
| **NCAM-180.** Official Name: Neural cell adhesion molecule 1; Official Symbol: NCAM1 (Neural cell adhesion molecule, large isoform precursor) | up: GGCGAGGACTTTAAAATGGAC | 263 |
|  | do: TTTGTCTGTGTGGCGTCATT |  |
| **Neurofilament-H.** Official Name: Neurofilament, heavy polypeptide; Official Symbol: NEFH | up: CTGGACGCTGAGCTGAGGAA | 316 |
|  | do: CAGTCACTTCTTCAGTCACT |  |
| **Neurofilament-L.** Official Name: Neurofilament, light polypeptide; Official Symbol: NEFL | up: TCCTACTACACCAGCCATG | 284 |
|  | do: TCCCCAGCACCTTCAACTTT |  |
| **Neurofilament-M.** Official Name: Neurofilament, medium polypeptide; Official Symbol: NEFM | up: CGAATACCAGGACCTCCTCA | 206 |
|  | do: TGTGTTGGACCTTAAGCTTGG |  |
| **NSE.** Official name: Homo sapiens enolase 2 (gamma, neuronal); Official Symbol: ENO2 | up: GGAGTTGGATGGGACTGAGA | 392 |
|  | do: GCTCCAAGGCTTCACTGTTC |  |
| **Runx2.** Official name: Homo sapiens runt-related transcription factor 2; Official Symbol: RUNX2 | up: CTCAGTGATTTAGGGCGCAT | 348 |
|  | do: CTGGCTCTTCTTACTGAGAG |  |
| **Synaptophysin.** Official name: Homo sapiens Synaptophysin; Official Symbol: SYP | up: CGAGGTCGAGTTCGAGTACC | 380 |
|  | do: TCAGCTCCTTGCATGTGTTC |  |
| **NTRK1.** Official name: neurotrophic tyrosine kinase, receptor, type 1; Official Symbol: NTRK1 | up: TGTTGGCAGCAAGCTACATC | 331 |
|  | do: CACTGCAGCTTCTGTTCAGG |  |
| **NTRK2.** Official name: Homo sapiens neurotrophic tyrosine kinase receptor type 2; Official Symbol: NTRK2 | up: CAATTGTGGTTTGCCATCTG | 345 |
|  | do: ACAGTGAATGGAATGCACCA |  |
| **NTRK3.** Official name: Homo sapiens receptor tyrosine kinase TrkC; Official Symbol: NTRK3 | up: AGCGTCTGGCTGGACTATGT | 367 |
|  | do: GTGTGGTGAGCCGGTTACTT |  |
| ß**III-Tubulin.** Official name: Homo sapiens tubulin, beta 3 class III; Official Symbol: TUBB3 | up: CGCCCAGTATGAGGGAGAT | 195 |
|  | do: CTCGAGGCACGTACTTGTGA |  |
