# Supplementary material for: Staurosporine and Extracellular Matrix Proteins Mediate the Conversion of Small Cell Lung Carcinoma Cells into a Neuron-Like Phenotype
Source: PLoS One. 2014 Feb 28;9(2):e86910. doi: 10.1371/journal.pone.0086910 (PMC3938400; doi:10.1371/journal.pone.0086910)
Supplement: Table S2 — Cell cycle analysis of SCLC cells: Quantification of cell viability, apoptotic and necrotic cell death. SCLC cells were either cultured in the absence (- FCS) or presence of fetal calf serum (+ FCS), or cultured in the presence of serum and treated for 24 h with 20 or 50 nM SSP or for 24 h with 50 nM SSP and then cultured for further 24 h in the absence of the drug (+/−) and analyzed by FACS. Subtables present data obtained for viable (AnnexinV− DAPI−; S2-1), apoptotic (AnnexinV+; S2-2) or necrotic (AnnexinV−, DAPI+; S2-3) cell populations. Values are given as percentages (mean values ± standard deviation) of the total number of events analyzed. Statistical difference to control cells cultured in the presence of serum (+ FCS) was assessed by unpaired t-test; *: p≤0.05. FCS: fetal calf serum, FIB: fibronectin; LAM: laminin; PO: polyornithine; SSP: staurosporine. (DOC) [file pone.0086910.s002.doc]

**Supplemental data Table S2 - Cell cycle analysis of SCLC cells**

**S2-1: Cell viability (AnnexinV^-^ DAPI^-^**):

| Cell line | SCLC | | H1184 | | GLC-2 | | GLC-36 | |
| --- | --- | --- | --- | --- | --- | --- | --- | --- |
| Treatment | PO | LAM | PO | FIB | PO | FIB | PO | FIB |
| - FCS | 69.93 ± 1.66 |  | 67.50 |  | 72.55 ± 7.57 |  | 84.50 ± 2.97 |  |
| + FCS | 67.48 ± 6.73 | 58.74 ± 12.80 | 82.98 ± 8.57 | 79.68 ± 8.57 | 70.67 ± 12.50 | 82.73 ± 6.18 | 79.46 ± 15.26 | 77.86 ± 15.99 |
| + FCS / 20 nM SSP | 68.47 ± 16.39 | 66.43 ± 16.49 | 77.07 ± 6.05 | 80.90 ± 6.15 | 75.20 | 80.50 | 61.37 ± 11.9 | 62.09 ± 11.17 |
| + FCS / 50 nM SSP | 58.30 ± 11.82 | 56.08 ± 12.81 | 78.22 ± 7.40 | 77.74 ± 4.42 | 80.95 ± 12.16 | 82.93 ± 11.81 | 61.60 ± 19.25 | 62.84 ± 16.01 |
| + FCS / +/- 50 nM SSP | 43.17 ± 11.15 * | 32.60 ± 17.38 * | 84.80 ± 14.57 | 81.20 ± 13.29 | 69.35 ± 7.42 | 74.70 ± 2.69 | 64.40 ± 10.09 | 63.83 ± 10.62 |

**S2-2: Apoptotic cells (AnnexinV^+^):**

| Cell line | SCLC | | H1184 | | GLC-2 | | GLC-36 | |
| --- | --- | --- | --- | --- | --- | --- | --- | --- |
| Treatment | PO | LAM | PO | FIB | PO | FIB | PO | FIB |
| - FCS | 7.45 ± 8.76 |  | 3.71 |  | 13.51 ± 14.8 |  | 4.69 ± 4.35 |  |
| + FCS | 21.75 ± 14.29 | 25.75 ± 17.61 | 3.51 ± 2.14 | 3.76 ± 3.66 | 14.40 ± 8.09 | 4.22 ± 1.05 | 4.55 ± 1.73 | 5.69 ± 2.55 |
| + FCS / 20 nM SSP | 25.24 ± 20.93 | 24.98 ± 22.38 | 6.15 ± 4.89 | 4.55 ± 5.66 | 15.94 | 12.45 | 13.42 ± 8.28 * | 15.57 ± 8.37 * |
| + FCS / 50 nM SSP | 33.23 ± 16.6 | 32.68 ± 16.25 | 3.62 ± 3.69 | 5.26 ± 5.58 | 10.63 ± 10.07 | 10.71 ± 11.89 | 13.84 ± 9.77 * | 14.54 ± 12.89 |
| + FCS / +/- 50 nM SSP | 50.34 ± 16.44 * | 60.3 ± 24.1 | 8.55 ± 11.31 | 10.15 ± 13.58 | 14.5 ± 8.3 | 15 ± 10.53 | 13.53 ± 6.51 * | 17.43 ± 8.81 * |

**S2-3: Necrotic cells (AnnexinV^-^ DAPI^+^**):

| Cell line | SCLC | | H1184 | | GLC-2 | | GLC-36 | |
| --- | --- | --- | --- | --- | --- | --- | --- | --- |
| Treatment | PO | LAM | PO | FIB | PO | FIB | PO | FIB |
| - FCS | 22.63 ± 8.70 |  | 28.7 |  | 13.96 ± 7.27 |  | 10.82 ± 1.39 |  |
| + FCS | 10.79 ± 8.38 | 15.54 ± 7.93 | 13.52 ± 3.55 | 16.52 ± 5.49 | 14.58 ± 9.58 | 13.05 ± 7.13 | 15.98 ± 14.85 | 16.46 ± 13.87 |
| + FCS / 20 nM SSP | 6.3 ± 4.72 | 8.59 ± 6.74 | 16.76 ± 10.68 | 14.57 ± 5.75 | 8.92 | 7.05 | 25.2 ± 7.01 | 21.53 ± 2.75 |
| + FCS / 50 nM SSP | 8.36 ± 7.09 | 11.19 ± 5.58 | 18.16 ± 5.98 | 17 ± 4.71 | 8.42 ± 5.93 | 6.37 ± 5.32 | 24.54 ± 12.78 | 22.62 ± 7.9 |
| + FCS / +/- 50 nM SSP | 6.49 ± 5.37 | 7.15 ± 6.75 | 6.64 ± 3.30 | 8.68 ± 0.30 | 18.02 ± 12.60 | 10.28 ± 7.93 | 22.1 ± 15.48 | 18.75 ± 19.21 |
